# Diminished neural responses for greater numbers of lives at risk and lives lost in foreign countries

**DOI:** 10.1093/scan/nsag049

**Published:** 2026-07-08

**Authors:** Daniel Campbell-Meiklejohn, Jo Cutler

**Affiliations:** School of Psychology, University of Sussex, Brighton, East Sussex, BN1 9QH, United Kingdom; Sussex Neuroscience, The University of Sussex, Brighton, BN1 9RH, United Kingdom; Sussex Centre for Research on Kindness, University of Sussex, Brighton, East Sussex, BN1 9RH, United Kingdom; School of Psychology, University of Sussex, Brighton, East Sussex, BN1 9QH, United Kingdom; Sussex Neuroscience, The University of Sussex, Brighton, BN1 9RH, United Kingdom; Centre for Human Brain Health, School of Psychology, University of Birmingham, Birmingham, West Midlands, B15 2TT, United Kingdom; Institute for Mental Health, School of Psychology, University of Birmingham, Birmingham, West Midlands, B15 2TT, United Kingdom; Centre for Developmental Science, School of Psychology, University of Birmingham, Birmingham, West Midlands, B15 2TT, United Kingdom

**Keywords:** bias, risk, death, compassion, scope insensitivity, empathy

## Abstract

Humanitarian crises fail to elicit responses proportional to their scale. Two psychological factors that may contribute to this problem are *scope insensitivity*, where concern fails to increase proportionally with the number of lives at risk, and *home country bias*. We studied these factors with functional magnetic resonance imaging (fMRI) and behavioural measurement. Twenty-five participants completed scanning while viewing ‘headlines’: summaries describing a specified number (from one to tens of thousands) of lives that were at risk in either their home country or abroad, followed by information confirming their survival or death. Control trials presented analogous real financial risks. Whereas neural responses to financial risk scaled positively with risk magnitude, responses to life risk declined with larger numbers of lives at home and followed an ‘inverted-U’ in response to lives abroad: low for single lives abroad, higher for moderate numbers, but also low for the greatest numbers. Deaths only evoked stronger responses than survival for home country victims, in neural regions previously associated with emotion derived from harm coming to other people. Independent *willingness to pay* ratings also demonstrated both biases. This study provides neural characterizations of scope insensitivity and home country bias when learning about lives at risk.

## Introduction

Few moral questions are as enduring as how we value a human life (Smith 1759, Singer 1972, Aristotle 2007). Across religious, political, and humanitarian traditions, a central principle is that one stranger’s life should be valued no less than another’s (e.g. United Nations 1948, International Committee of the Red Cross 2016). Yet, global inequalities in life expectancy (United Nations Development Program 2025), declining foreign aid (Organisation for Economic Cooperation and Development 2025), and chronic shortfalls in humanitarian assistance (United Nations Office for the Coordination of Humanitarian Affairs 2025) indicate that lives, in practice, are not valued equally.

Among the many complexities of saving lives in humanitarian crises, funding and political will can be constrained by the extent to which lives at risk and prior fatalities elicit a response in those with the power to determine future outcomes (Slovic 2007, Batson 2010, Evangelidis and Van den Bergh 2013, Lerner *et al.* 2015, Olivola 2015, Lee and Feeley 2016, Arcos González and Gan 2024). Two features of humanitarian disasters can downgrade this response: they impact very large numbers of people, and they typically occur in foreign countries. Both effects have been examined across philosophy, psychology, and media studies; here, we examine them through a neural lens.

A failure to scale affective responses with the number of people at risk is an instance of *scope insensitivity* (Dickert *et al.* 2015, Chang and Pham 2018). Different functions have been proposed to capture the value of saving lives across different numbers of unidentified victims (e.g. Slovic 2007, Dickert *et al.* 2012, Slovic and Västfjäll 2015), any of which could be associated with an underlying neural response. One is a concave function, termed ‘psychophysical numbing,’ in which the value of life-saving action *increases, but at a diminishing marginal rate* with the number of lives at risk (Fetherstonhaugh *et al.* 1997). This is analogous to diminishing sensitivities associated with increasing expected utility (Bernoulli 1954, Kahneman and Tversky 1979) and sensory stimulation (Fechner 1860). Alternatively, neural responses could reflect an overall ‘collapse of compassion’ where engagement *decreases* with the number of lives at stake. This might occur when processes evoked by just a few lives (e.g. empathy) are replaced with statistical thinking for larger numbers (Small *et al.* 2007, Dickert *et al.* 2015). These two patterns have also been proposed to combine into an *‘inverted-U’*, with engagement rising at lower numbers before collapsing at higher magnitudes of potential loss (Västfjäll *et al.* 2014, Slovic and Västfjäll 2015). Guided by these proposals, we explored how neural responses to life‑risk information vary with the number of lives affected. Potential financial losses were also tested as a non-social reference condition for which we expected positive scaling with magnitude in neural regions tracking salience and anticipatory threat (Berns *et al.* 2006, Bartra *et al.* 2013).

People also engage more with news about their home country (Morton and Warren 1992, Aalberg *et al.* 2013, Mitchell *et al.* 2018) and prefer to donate to domestic causes (Micklewright and Schnepf 2009, Casale and Baumann 2015, Guéguen *et al.* 2018, Cutler *et al.* 2021). This *home country bias* may be because national borders coincide with factors known to shape moral evaluation and the perceived identity, similarity, threat, and social distance of those who need help (e.g. Levine *et al.* 2005, Mittelman and Dow 2018, Zagefka 2018, Chapman *et al.* 2025), which in turn may alter neural responses to their situation (e.g. Mobbs *et al.* 2009, Xu *et al.* 2009, Hein *et al.* 2010, Cikara and Van Bavel 2014, Strombach *et al.* 2015, Tusche *et al.* 2016). We therefore sought to determine whether neural responses to loss of life reflect a greater affective response when deaths occur in one’s home country. In addition to whole brain analyses, we examined three *a priori* regions of interest (ROIs) within bilateral anterior insula and dorsomedial prefrontal cortex derived from a meta-analysis of vicarious pain. These regions are proposed to host socially derived emotional processes that can be modulated by perceived characteristics of the person being observed (e.g. Singer *et al.* 2006, Craig 2009, Xu *et al.* 2009, Hein *et al.* 2010, Bruneau *et al.* 2012, Cikara and Van Bavel 2014), rather than a low-level sensory experience of pain (Lamm *et al.* 2011). These regions also correspond to neural correlates of the most salient changes in subjective value (Bartra *et al.* 2013). We predicted that heightened affective responses to the permanence and greater harm associated with death relative to survival would be expressed in these areas and modulated by the victim’s country.

**Figure 1:**
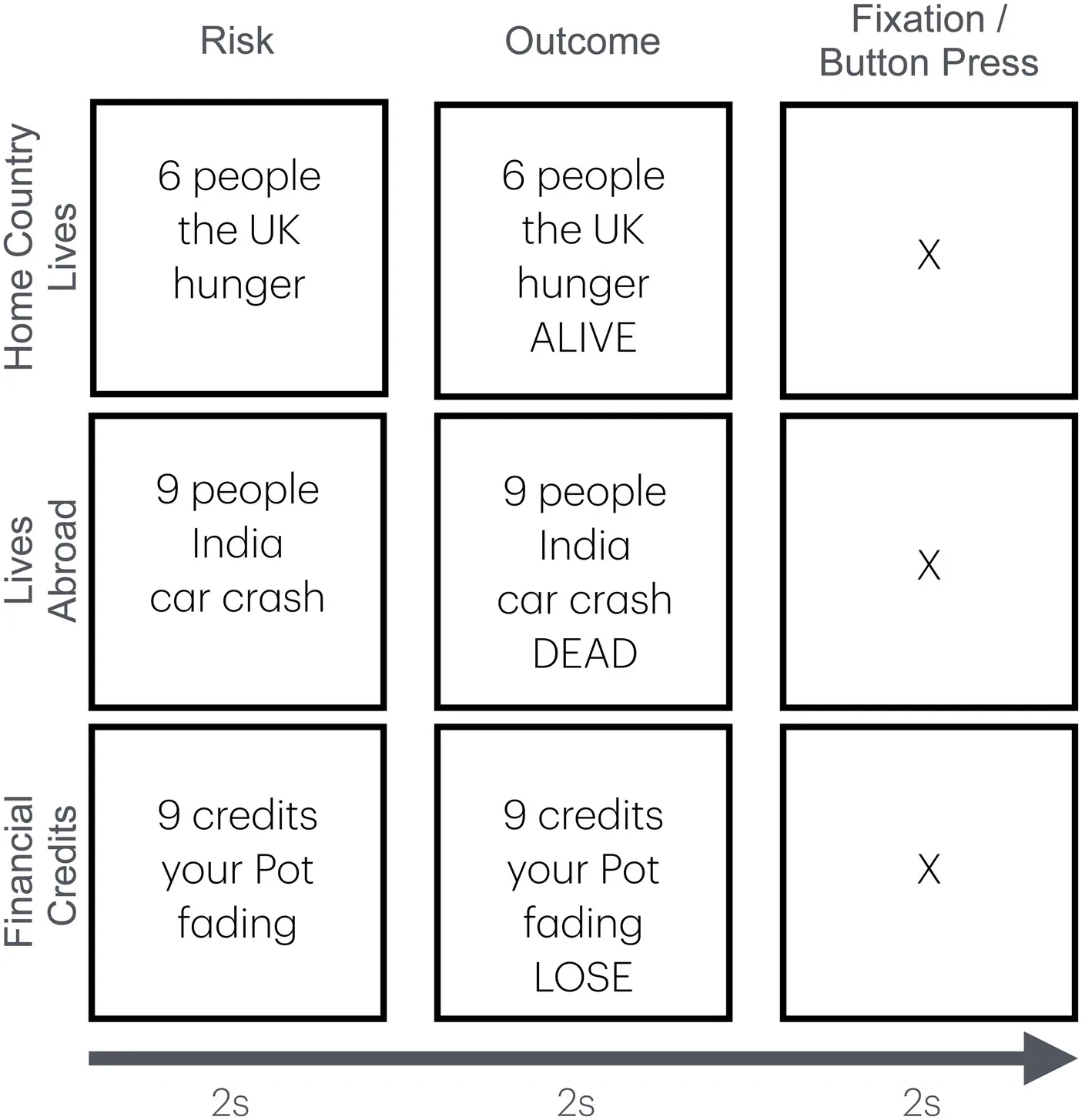
The Headlines Task with examples of home country lives, lives abroad, and financial credit trial types. Life risk trials were presented in the form: [number] people, [country], [cause]. Financial control trials were presented in the form: [number] credits, ‘Your Pot’, [cause]. Outcomes were presented below the headline, followed by a fixed intertrial interval that included a request for a button press.

A ‘*Willingness to Pay’* task was used to assess self-reported behavioural scope insensitivity and home country bias through hypothetical financial allocations to life-saving causes. We then used our new ‘*Headlines Task*’ (Fig. 1) with fMRI, including the non-social control, to explore the neural correlates of scope insensitivity and home country bias. In this task, participants read 200 ‘headlines’ about one to tens of thousands of real people who were at risk of death, either in the participant’s home country or one of 56 foreign countries, followed by information on whether they lived or died.

## Methods

### Participants

Twenty-six healthy participants took part. This number was set by our funding and determined to be adequate for testing social-affective response modulation at the time of data collection based on prior studies (Bartra *et al.* 2013 for review; Lamm *et al.* 2007, Hein *et al.* 2010, Genevsky *et al.* 2013, Campbell-Meiklejohn *et al.* 2017, Szucs and Ioannidis 2020). No data were analysed prior to all data being collected. One participant was excluded due to scanner issues, leaving 25 participants (12 female; age M = 26.56, SD = 5.35, range 20–37). All participants self-identified as being right-handed. Twenty-four of the included participants identified as British and one identified as Irish. All were UK residents and lived most of their lives in the UK. Exclusion criteria determined by interview were standard MRI safety exclusions (any implanted medical device, heart surgery, recent surgery of any kind, any metal in the body, epilepsy, skin patches, tattoos in the upper part of the body, or chance of pregnancy), dyslexia, dyscalculia, current physical illness, history of head injury, current mental health issues or addictions, or having studied psychology, economics, mathematics, or physics at degree level. Participants were paid £30. Voting tendencies were recorded to understand the political leanings of our sample, which may be associated with differences in globalist or isolationist preferences that influence interest in lives abroad. When asked who they would vote for if a general election were tomorrow, 12 answered Green, 6 Labour, 3 Liberal Democrat, 2 didn’t say, and 2 would not vote. All but two reported voting ‘remain’ in the Brexit referendum (1 did not vote, 1 preferred not to say). The study was approved by the Brighton and Sussex Medical School Research Governance and Ethics Committee [ER/JC620/5]. All data were collected in 2019, before the COVID pandemic.

### Task & measures

#### Willingness to pay

We adapted an existing willingness to pay measure (Frederick and Fischhoff 1998). Participants were asked to imagine they were a UK government minister in charge of allocating resources to stop people dying from flooding both in the UK and abroad for 12 projects, 6 in the UK and 6 in named foreign countries, to save different numbers of people, with a £100 000 possible budget per project. Instructions can be found in the supplemental material, but to mitigate social desirability bias, instructions stated: ‘You must not spend unnecessary money because the money can also go to other worthy causes. If a project is not worthwhile, you can decide to give it £0.’ For example: ‘Project Z will save 10903 people in the Philippines from dying from flooding. Of £100 000 available for this project, I give ___.’ The numbers of lives to be saved per country were UK 1, 5, 32, 274, 5 273, and 10 812; Nepal 1; Myanmar 6; Nicaragua 35; Venezuela 268; Pakistan 5 291; and the Philippines 10 903.

For analysis, we used a linear mixed effects model predicting the amount allocated from (log_10_) number of people, location (home country or abroad, effects coded), and their interaction, together with a random intercept and a random slope for (log_10_) number of people.

#### Headlines task

Stimuli for the Headlines Task were adapted from real news stories, statistics, and online reports, in which varying numbers of people were at risk of dying in the home country (the United Kingdom) and abroad (56 different countries, Table S1). Each story was summarized as a ‘headline’ with the number of people, country, and cause of death. The five broad cause types were natural disasters, accidents, violence, diseases, or other long-term issues (e.g. hunger, poverty). Participants were informed before the task that, across headlines, half of the people would die and half would survive. Twenty conditions were formed according to a 5 (*number bins*) × 2 (locations: *home* or *abroad*) × 2 (*outcomes: positive:* alive or *negative*: dead) design. There were 10 headlines in each condition (200 headlines in total). Number bins were: 1, 2–10, 11–50, 51–500, and 501+ person(s)/credit(s). Broad cause types appeared equally across number bin and location conditions. Number bin and location conditions were also balanced for ratings of evoked emotion strength, evoked emotion valence (positive or negative) and experienced empathy from the specific causes of threat to life, using ratings from independent pilot participants (see supplemental material for full task development procedure).

Financial control trials involved a risk of losing credits, which represented entries into a lottery to win one of two £50 prizes. Participants started with 1 000 000 credits in their ‘pot’, but on each trial, some could be lost. We informed participants that there was a 50:50 chance of keeping or losing the trial’s credits. These 100 financial trials were in 10 conditions with a 5 (*number bin*) × 2 (*outcome: positive:* keep or *negative*: lose) design, with 10 trials per condition. To match the variation of different causes of death, credits also had different causes of being lost: fading, decaying, disappearing, destruction, or theft.

This created 30 conditions in total (see Table S2). The 200 headlines and 100 financial trials were intermixed. Trial order was set by dividing the 300 trials into 10 blocks. Each block had one randomly selected trial from each of the 30 conditions. The order of conditions within each block was then randomized, as was the order of blocks. This sequence was presented with no breaks between blocks. The headline without the outcome was presented for 2 s, followed by adding the outcome for 2 s–either ‘ALIVE’ or ‘DEAD’ for lives and ‘KEEP’ or ‘LOSE’ for credits (Fig. 1). Participants were then asked to indicate whether a fixation cross, presented for 2 s, was a + or a × by pressing one of two buttons on each trial.

#### Post-scan behavioural measures

After scanning, participants rated all life headlines with outcomes in random order, on two 0–100 scales. One measured surprise: ‘In the scanner, how surprising did you find this?’ [Not at all surprising – Very surprising] and the other emotion: ‘In the scanner, how did you feel about this?’ [Very negative – Very Positive]. See supplemental material for analysis.

We also asked participants whether there were any headlines for which they had a personal link to either the country or the cause.

Finally, participants answered a series of debriefing questions about their feelings of identity with their home country, the emotional impact of events happening to other people in different countries, worry about these events, priority of helping home or abroad countries, and attributions of home country bias (Table S3).

No participants reported not believing the headlines.

### Procedure

Following informed consent and safety checks, participants received instructions for the task in the scanner, including example trials. Stimuli were presented in MATLAB (2018a, MathWorks, Inc., Natick, MA) using Psychophysics Toolbox extension on a screen viewed via a mirror mounted on the head coil. Participants categorized the fixation cross using two buttons.

### Statistical analysis

All linear mixed models of this study used the *lme4 (v.1.1-38)* and *lmerTest (v.3.2-0)* packages in *R (v.4.5.2)*. *P* values for fixed effects were calculated using t-tests with Satterthwaite’s approximation for degrees of freedom.

### fMRI data acquisition

Data were acquired using a Siemens Prisma 3 T scanner (Siemens Medical Solutions, Erlangen, Germany) fitted with a 32-channel head-coil. Field maps were collected in the anterior–posterior and posterior–anterior directions to adjust for distortions. Functional T2-weighted echo-planar images (EPI) were acquired in an interleaved order with multiband factor 4 at a 30° angle to AC-PC. Each volume contained 52 slices with a TR of 1.5 s, TE of 37 ms, 52° flip angle, and voxel size of 2.19 × 2.19 × 2 mm. Finally, whole brain high-resolution T1-weighted structural images were collected using the MPRAGE sequence with the following parameters: 208 slices, 0.8 mm isotropic voxel size, 2.4 s TR, 2.22 ms TE, 8° flip angle.

### fMRI analysis

We used FMRIB’s Software Library (FSL, version 6.0.5).

#### Preprocessing

We used the default FSL preprocessing pipeline with linear registration of motion-corrected EPI images to an expanded functional image (full search, 6 DOF) and then to the participants’ structural image (full search, BBR). Registration of the structural to standard MNI space used nonlinear registration (full search, 12 DOF). Distortion correction was completed using fieldmaps prepared by FSL’s topup tool. Data were smoothed with a 5 mm full-width half-maximum Gaussian kernel. We further generated confound matrices (volumes to be ignored) for events of extreme motion based on root mean squared intensity difference of volume *N* to volume *N* + 1, thresholded at the 75th percentile + 1.5 times the interquartile range. Two participants with excessive motion toward the end of their scan had datasets truncated.

#### First level fMRI analysis

Separate general linear models (GLMs) were used to analyse responses to information associated with (a) risk presentation and (b) outcomes. Both GLMs used regressors of 2 s duration, with onsets timed to the relevant trial event (presentation or outcome onset). All regressors and their temporal derivatives were convolved with FSL’s default hemodynamic response function (gamma, delay: 6 s, SD: 3 s).

##### Presentation of risk

The first GLM consisted of a regressor for each of: home country life risk headline onsets and their temporal derivative, parametric home country life risk magnitude (i.e. weighted by risk magnitude), abroad life risk headline onsets and their temporal derivative, parametric abroad life risk magnitude, credit risk onset and their temporal derivative, and parametric credit risk magnitude. Since a linear relationship scaling from 0 to 50 000 lives or credits is implausible, we used the base-10 logarithm of the number of lives (or credits) as a continuous parametric predictor; a significant linear effect therefore indicates a concave response function where each additional life or credit has less effect than the last. In addition, we included 24 standard motion regressors, motion confound regressor matrices (see preprocessing), and a confound regressor of headlines to which participants reported a link to the cause or country (and removing these events from regressors of interest). Contrasts captured parametric effects in each condition (home country lives, lives abroad, financial credits). To test where scaling trajectories diverged based on the social versus financial nature of the risk, we computed direct parametric contrasts (interactions) between conditions.

To visualize these effects, we ran a separate GLM in which the number of lives/credits was divided into the five number bins used in the task design. This GLM modelled 15 conditions–number bin (1–5) × trial type (home, abroad, credits)—each containing 20 trials, alongside 24 motion regressors and confound regressors as described above. We then extracted the mean parameter estimate within each condition for each cluster of interest for plotting purposes. Guided by prior literature and these visualizations, we also conducted *post hoc* whole brain contrasts.

##### Survival and death

This GLM of outcomes consisted of thirty primary regressors and temporal derivatives timed to the outcome onset. These covered each condition combination from *five number bins* (*1–5*), three *trial types* (*home country*, *abroad*, or *credits*) and two *outcome valences* (*alive/keep* or *dead/lose*). In addition, we included 24 standard motion regressors, motion confound regressor matrices (see preprocessing), and a confound regressor of headlines to which participants reported a link. This bin-level structure allowed risk magnitude to enter the ROI analysis (see below). Contrasts were made between positive (alive/keep) and negative (dead/lose) outcomes within each trial type (home country lives, lives abroad, or financial credits) and interaction effects between outcome and trial type.

#### Whole brain second level analyses

Group-level models were fitted to parameter estimates from each of the whole brain lower-level analyses, using FMRIB’s Local Analysis of Mixed Effects (FLAME) 1 + 2. We used cluster-corrected thresholding with a voxel threshold of *Z *> 3.1 and cluster threshold of *P* < .05, unless otherwise indicated. Statistical maps are overlaid onto the MNI 152 brain with MNI coordinates of the slice indicated in mm. Locations of peak responses were identified using the Harvard-Oxford cortical and subcortical atlases.

To test for spatial overlap of effects across conditions, we performed conservative conjunction analyses using the minimum Z statistic (*Z *> 3.1, *P <* .05) (Nichols *et al.* 2005).

#### Region of interest analyses

For the analysis of differential responses to positive and negative outcomes (alive/keep, dead/lose), we created a priori regions of interest (ROIs) for processing outcomes. We used peaks identified in an fMRI meta-analysis as consistently responsive to socially derived emotions from vicarious pain across 32 studies (Lamm et al. 2011). These peaks are within areas also affected by the most salient changes in subjective value (Bartra et al. 2013). These were: dorsomedial prefrontal cortex (dmPFC) (MNI coordinates of centre: *x* = −2, *y* = 23, *z* = 40); left anterior insula (−40, 22, 0); and right anterior insula (39, 23, −4). For each, we extracted average parameter estimates from a 5 mm sphere for each of the 30 conditions. We then started with a maximal model predicting the average parameter estimates of each ROI from the mean-centred log10 of the average number (people/credits) associated with number bin, outcome (alive, dead), location (home country or abroad) and all possible interactions as fixed and random effects. Using the *buildmer R package (v.2.12)*, we found the maximal model that converged and performed backward stepwise elimination using likelihood ratio tests. All three final models included all main effects, interactions of outcome × location and outcome × number, and a random intercept. Factors were effects coded.

## Results

### Explicit valuation of lives at risk

**Figure 2:**
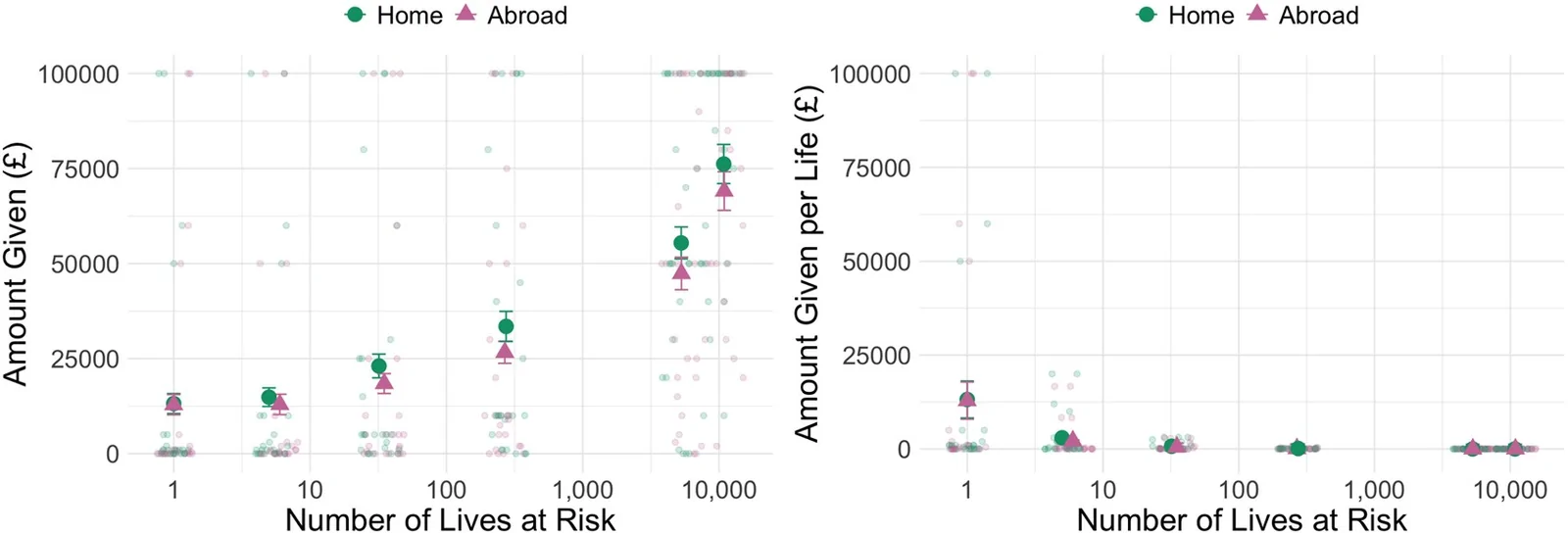
Willingness to Pay. Hypothetical amounts given to save groups of different sizes (log_10_ scale) suggest home country bias and diminishing value per life as the number of lives at risk increases. Error bars are within-subject standard error.

For the willingness to pay task (Fig. 2), participants spent more money to save lives in their home country (*M* = £36 035 total, *M* = £2834 per person) than to save lives abroad (*M* = £31 237 total, *M* = £2614 per person), *B *= −5060, *SE *= 1945, *t*(248) = −2.60, *P* = .010. Spending scaled logarithmically with the number of people, with diminishing marginal value for each additional life, *B *= 13 809, *SE *= 1586, *t*(24) = 8.71, *P* < .001. Average per-person allocations were high for a single life (£13 175 home, £12 884 abroad) but far lower for individuals in groups of over 10 000 (£7.04 home, £6.33 abroad). Importantly, this diminishing per-person pattern was already evident at small numbers, where the £100 000 cap was rarely reached: per-person allocation fell from £13 175 at one home country life to £2972 at five lives and £721 at thirty-two lives, with only 2–3 of 25 participants choosing the maximum £100 000 in any of these conditions. The location by number interaction was not significant (*P* = .194).

### Neural responses to lives at risk show patterns of scope insensitivity depending on their country

**Figure 3:**
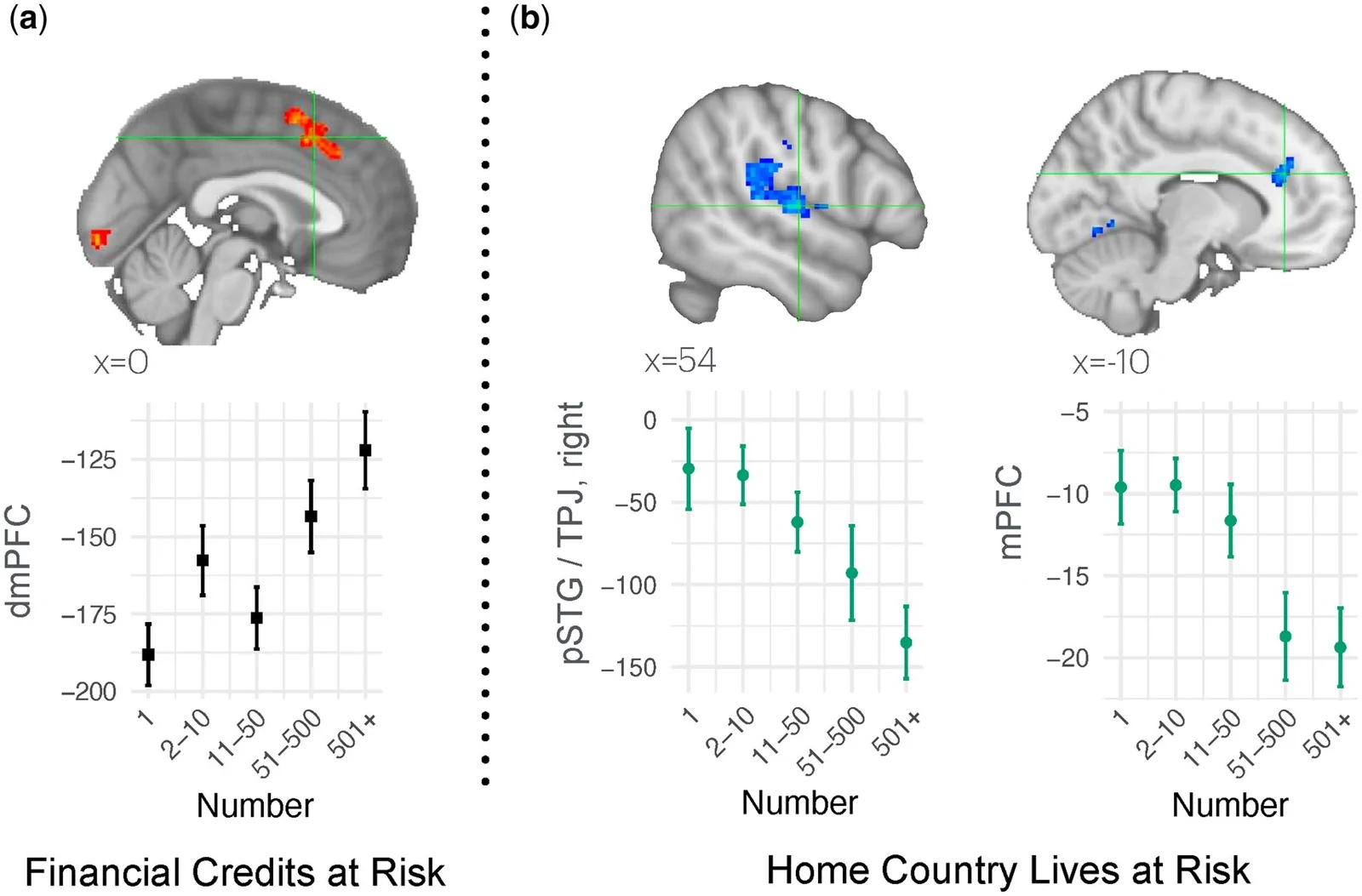
Neural Response Changes with Number at Risk. Statistical maps show significant clusters of linear parametric responses to the (log_10_) numbers of: (a) financial credits (increasing) and (b) home country lives (decreasing) (*Z *> 3.1, cluster *P* < .05). Plots below display average parameter estimates within each cluster, for each number bin. These are for visualization purposes only. Error bars are within-subject standard error. See Figs S1 and S2 for all significant clusters.

We examined where activity was modulated by risk magnitude for: home country lives, lives abroad, and financial credits (Fig. 3). Neural responses *increased* logarithmically with higher numbers of credits in visual processing areas and dmPFC (Figs 3a and S1A, Table S4). No responses were inversely related to the magnitude of potential loss of financial credits.

Strikingly, unlike the effect of financial risk magnitude, there were no brain regions where activity increased with the number of people at risk in the home country (Figs 3b and S2, Table S5). Responses consistently *decreased* for greater numbers of lives at risk in many regions spanning the posterior superior temporal gyrus and temporoparietal junction (pSTG/TPJ), medial prefrontal cortex (mPFC), occipital cortex, lingual gyrus, and central opercular cortex.

**Figure 4:**
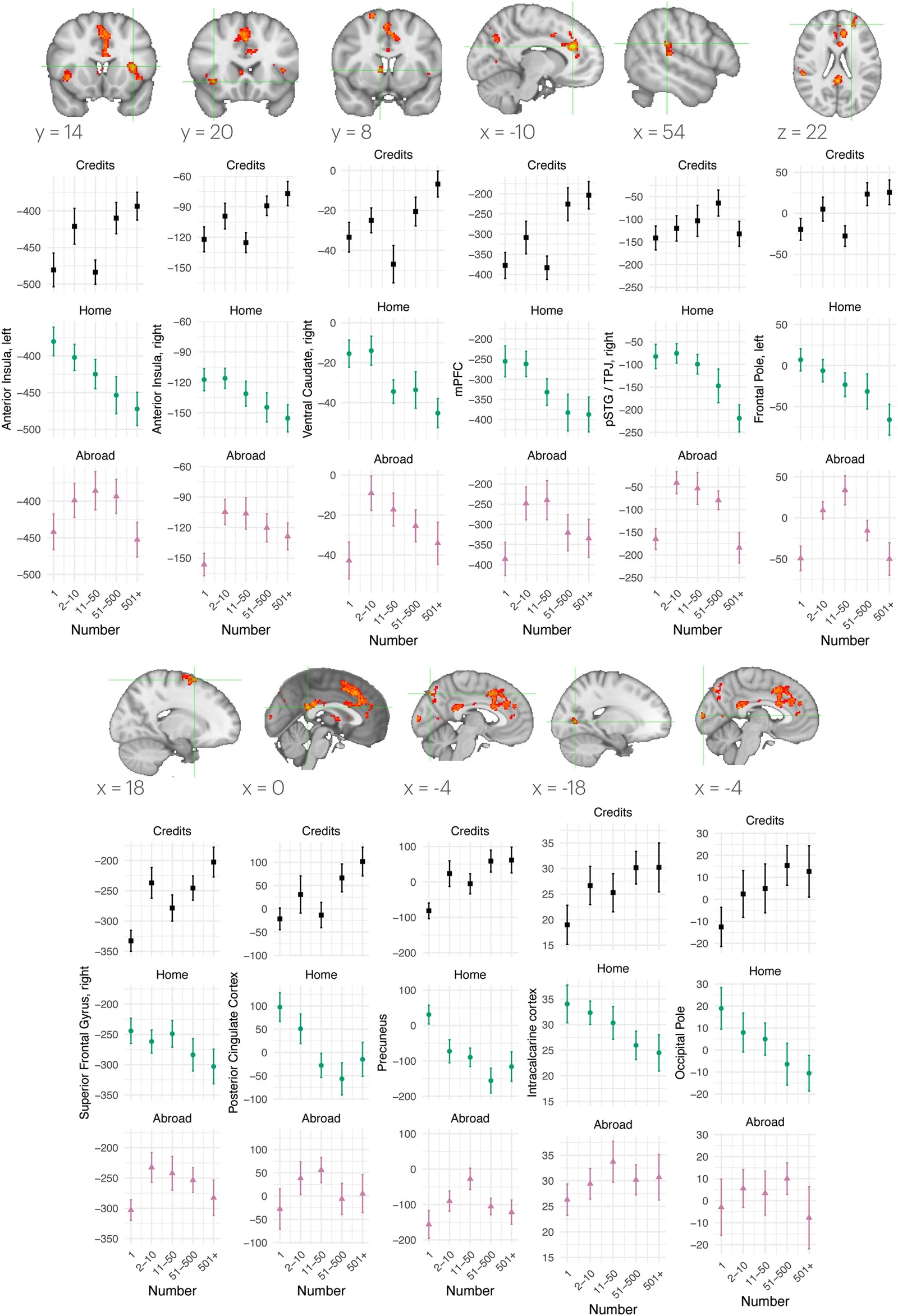
Differences in the neural response to the number at risk between home country lives and financial credits. Statistical maps show where the parametric response to the (log_10_) number at risk differed between home country lives and financial credits (*Z *> 3.1, cluster *P* < .05). Plots below display average parameter estimates within each cluster, for each number bin. These are for visualization purposes only. Error bars are within-subject standard error. Responses to the number of lives at risk abroad within these clusters are also shown. Home = green circles; abroad = pink triangles; credits = black squares. Images are in radiological (R-L) orientation.

We directly contrasted responses to risk magnitude between financial credits and home country lives, identifying eleven clusters where the direction of sensitivity to loss magnitude differed significantly (Fig. 4, Table S6). Visualization showed these effects involved overall *decreasing* responses for greater numbers of lives, contrasting with *increasing* responses for greater numbers of credits in the same regions. Although these regions distinguished magnitude sensitivity for the potential loss of home country lives versus financial credits, their independent responses to risk magnitude showed no conservative conjunction, suggesting that life-risk and financial-risk processing also rely on partly non-overlapping regions.

**Figure 5:**
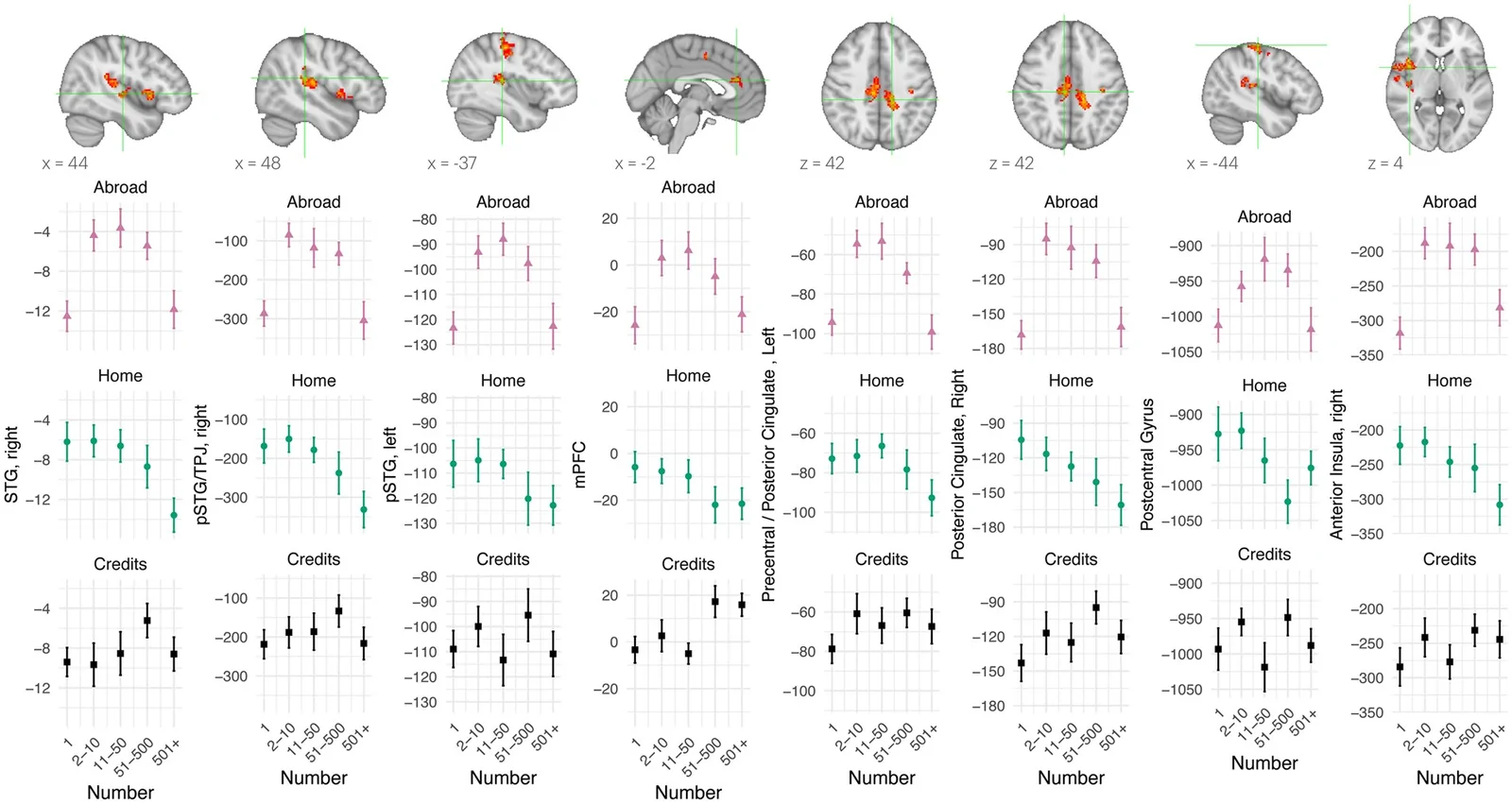
Inverted-U response to the number of lives at risk abroad. Statistical maps show where the response to 2–500 lives abroad was greater than for 1 and 501+ lives (*Z *> 3.1, cluster *P* < .05). Plots below display average parameter estimates within each cluster, for each number bin. Responses to the number of home country lives at risk and financial credits within these clusters are also shown. These are for visualization purposes only. Error bars are within-subject standard error. Home = green circles; abroad = pink triangles; credits = black squares. Images are in radiological (R-L) orientation.

For lives abroad, responses increased logarithmically with risk magnitude in occipital cortex. However, visual exploration of neural responses scaling with greater numbers of lives at risk compared to financial credits indicated a distinct inverted-U pattern for lives abroad across most responsive regions (Fig. 4), with an initial increase in response from one to small numbers of lives and a decline for very large numbers. Guided by predictions of an inverted-U in prior literature and this observation, we ran a whole brain *post hoc* analysis that explicitly tested for peaks in middle ranges of lives abroad (an inverted-U: with +1/3 weighting for the three middle bins and −1/2 weights for individual and 501+ lives). This shape effectively captured peaks in the three mid ranges relative to very low and very high numbers of lives and coincided with significant clusters in pSTG/TPJ, anterior insula, posterior cingulate and mPFC (Fig. 5, Table S7).

A conservative conjunction map (thresholded at *Z *> 3.1, *P <* .05) of the minimum *Z* values between the declining linear effect for home country lives and the inverted-U pattern for lives abroad, revealed that the right pSTG/TPJ (223 voxels, *Z*_max_ = 3.89, *P* = .012, *x* = 52, *y* = −26, *z* = 20) is robustly sensitive to the number of lives at risk across both location conditions. Across number bins, the primary difference between lives at home and lives abroad appeared to be for single lives at risk. A *post hoc* whole brain contrast between single lives at home and single lives abroad confirmed lower responses to single lives at risk abroad in seven clusters, including the right posterior TPJ, mPFC, posterior cingulate, inferior frontal gyrus, and precuneus (Table S8).

At the presentation of life risk, the main effect of location, while controlling for number of lives, showed greater activity in right orbitofrontal gyrus, bilateral temporal fusiform gyrus, precuneus, and occipital fusiform gyrus if lives were at risk abroad (Fig. S3, Table S9).

### Neural responses demonstrate home country bias in the processing of death and survival

**Figure 6:**
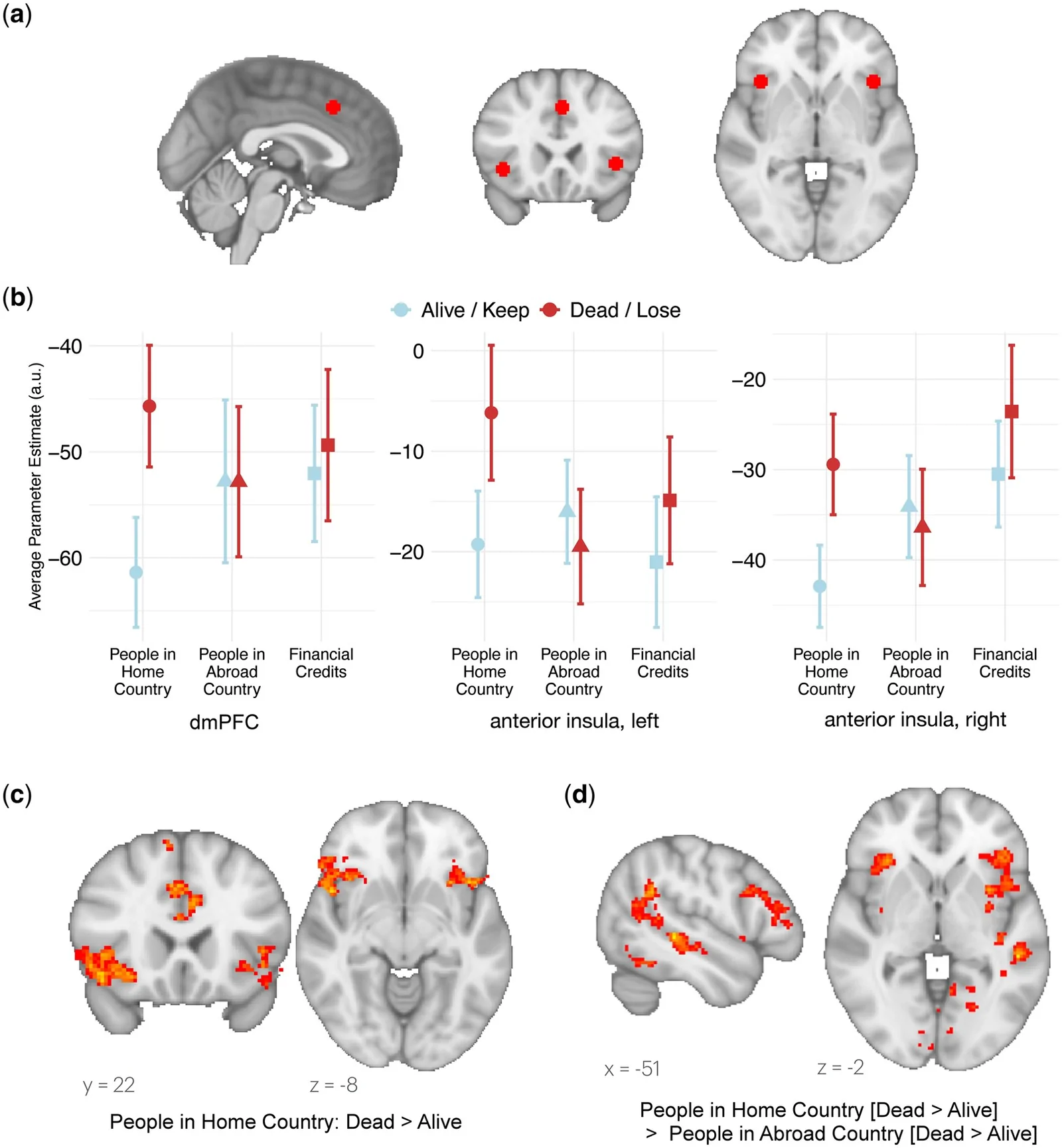
Neural responses to negative (dead/lose) versus positive (alive/keep) outcomes. (a) Regions of interest map. (b) Parameter estimates for negative and positive outcomes within the ROIs for lives and financial credits. Error bars represent within-subject 95% confidence intervals for statistical inference. (c) Statistical maps of clusters with greater activity for Dead than Alive outcomes in the home country in whole brain search (*Z *> 3.1, cluster *P* < .05). (d) Statistical maps of clusters associated with the Location [Abroad > Home Country] × Outcome [Dead > Alive] interaction in whole brain search (*Z *> 2.3, cluster *P* < .05).

We next compared the neural responses to life-risk outcomes between location conditions. Focusing on the relative response of *dead vs alive* ensured effects were not driven by general differences between location conditions (*home vs abroad*). From a mixed linear regression (Tables S10, S11, S12) we observed a main effect of ‘dead’ compared to ‘alive’ in each ROI (Fig. 6a): dmPFC *t*(490) = 2.25, *P* = .025 and right anterior insula *t*(490) = 2.09, *P* = .037, with a trend in the left anterior insula *t*(490) = 1.70, *P* = .090.

However, we also observed critical interactions. This effect of death was significantly lower if it occurred abroad (vs home), in all three regions: dmPFC *t*(490) = −2.26, *P* = .024, right anterior insula *t*(490) = −2.95, *P* = .003, left anterior insula *t*(490) = −2.92, *P* = .004 (Fig. 6b). Only home country headlines generated a neural response that distinguished between death and survival in these regions. The left anterior insula interaction was driven by a stronger response to deaths at home than deaths abroad. In the dmPFC and right anterior insula, no single effect drove the interaction (Table S13).

The response to *dead vs alive* increased with the number of victims in the dmPFC *t*(213) = 2.56, *P* = .011, right anterior insula *t*(213) = 2.15, *P* = .032, and left anterior insula ROI, *t*(213) = 2.00, *P =* .046. This was consistent across location conditions.

A whole brain analysis of *dead vs alive* in the home country confirmed enhanced response to loss of life in our regions of interest, with the addition of the hippocampus, mPFC, and lateral orbitofrontal cortex (Fig. 6c, Table S14). The whole brain analysis also found the interaction between location and outcome was significant at a slightly relaxed voxel threshold in clusters encompassing bilateral anterior insula, frontal operculum, and lateral orbitofrontal cortex (reported at *Z* > 2.3 cluster-corrected at *P* < .05 because bilateral effects and ROI analyses provide reassurance that these clusters did not emerge by chance) (Fig. 6d, Table S15).

### Emotion, surprise, and rationale for home country bias

After the task, participants rated headlines as relatively more emotionally positive when they described survival, fewer affected people, and events occurring abroad. Partial interactions showed death and victim number influences on emotion were greater, on average, if the lives were in the home country, but a three-way interaction was also present (Table S16).

Larger numbers of lives affected and events at home were rated as more surprising, with interactions showing that the effect of larger numbers of lives on surprise was greater for home country events, and the effect of death on surprise was less for lives abroad (Table S17).

Finally, participants attributed home country bias more to the likelihood that UK events would affect themselves *t*(24) = 2.52, *P* = .019 or someone they knew *t*(24) = 2.59, *P* = .016, compared to identification with victims (see Table S3 for mean ratings).

## Discussion

Neural and behavioural results were consistent with scope insensitivity and home country bias.

Behaviourally, participants provided lower allocations to causes in foreign countries and less money per person for larger numbers of victims, consistent with prior research (Fetherstonhaugh *et al.* 1997, Frederick and Fischhoff 1998, Slovic 2007, Micklewright and Schnepf 2009, Casale and Baumann 2015, Dickert *et al.* 2015, Guéguen *et al.* 2018, Cutler *et al.* 2021).

Neural responses to financial credits increased with potential loss magnitude in regions known to track salience and anticipatory threat (Berns *et al.* 2006, Bartra *et al.* 2013). Responses to home country lives showed a very different pattern: no regions scaled positively with number, while widespread decreases were present across right pSTG/TPJ, paracingulate gyrus, central opercular cortex, and visual areas. A direct contrast confirmed that the direction of magnitude sensitivity in eleven regions depends on whether lives or financial outcomes are at risk (Fig. 4), including regions associated with arousal, emotion, and value (anterior insula, mPFC, ventral caudate) (e.g. Craig 2009, Lamm *et al.* 2011, Bartra *et al.* 2013), social cognition (right pSTG/TPJ) (e.g. Decety and Lamm 2007), self-referential processing (precuneus, posterior cingulate) (e.g. Northoff *et al.* 2006) and low-level visual processing. The absence of a conjunction between the peak magnitude effects of financial risk and home country lives, however, suggests that the number of lives at risk not only drives neural responses in the opposite direction to the magnitude of financial risk; it also involves partially distinct neural regions. Overall, the neural pattern is consistent with a ‘collapse of compassion’ for larger numbers of home country lives, but did not reflect ‘psychophysical numbing (Dickert *et al.* 2012, 2015, Slovic and Västfjäll 2015).’

In these same regions, an inverted-U pattern was observed across numbers of lives at risk abroad (Fig. 4) and formally confirmed via whole brain search (Fig. 5). Responses collapsed for the largest numbers of lives abroad, as for home country lives, but responses to individual lives at risk abroad were also low–a pattern absent for the home country condition. A direct contrast confirmed that responses to individual lives depended on location. The inverted-U is consistent with proposals describing an initial increase in the subjective value of saving lives, followed by eventual collapse at larger numbers (Västfjäll *et al.* 2014, Slovic and Västfjäll 2015). The low response to individual lives abroad may additionally reflect relative underexposure to single-victim stories from foreign countries in media (Olivola and Sagara 2009). If so, identifying individual victims abroad may be particularly effective at increasing the subjective value of helping them (e.g. Genevsky *et al.* 2013, Lee and Feeley 2016).

A conjunction analysis identified right pSTG/TPJ as consistently sensitive to the number of lives in both the home country and abroad, suggesting that limits on perspective-taking and social imagery–not feasible for very large numbers in any country, and potentially challenging if individual victims are abroad–may be a common mechanism underlying scope insensitivity across geographical locations (Decety and Lamm 2007).

Deaths in the participant’s home country evoked greater neural responses than survival in brain regions associated with socially derived affect and salient changes in subjective value (Craig 2009, Lamm and Singer 2010, Lamm *et al.* 2011, Bartra *et al.* 2013). This effect of deaths relative to survivals was greater for lives at risk in the home country compared to lives at risk abroad (which showed no difference between death and survival). These findings extend prior evidence that victim identity modulates empathy-related neural responses (Xu *et al.* 2009, Hein *et al.* 2010, Bruneau *et al.* 2012, Azevedo *et al.* 2013, Cikara and Van Bavel 2014, Feng *et al.* 2016), to the critical context of complete loss of life across national borders.

National borders coincide with many perceived characteristics of victims, including culture, race, language, religion, politics, economic development, education, physical distance, and colonial ties (Mittelman and Dow 2018). Home country bias is likely to be an instantiation of in-group, distance, and related effects on concern for others (Chapman *et al.* 2025, Levine *et al.* 2005, Strombach *et al.* 2015, Zagefka 2018). Similar mechanisms may also drive differential responses to lives at risk among people with different identities within the same country–an important direction for future work. However, the physical proximity of threatening health news also affects autonomic response and engagement (Wise *et al.* 2009), suggesting home country deaths may be processed uniquely because they are more likely to affect the self or someone they know (Aristotle 2007, Zagefka 2018). Self-ratings supported this. The likelihood of events happening to oneself or people one knows was rated by participants as more likely to underpin home country bias than identification with victims. Supplemental analyses suggested home country bias may also relate to risks abroad being less surprising and less inductive of negative emotion than similar risks at home.

The overall decline of neural response for higher numbers of lives differed from the diminishing marginal pattern of fund allocations, which showed a concave increase in total spending with number. More research is needed to understand this difference fully, but neural declines may contribute to the reduced per-person allocations for large numbers while other decision-making processes mitigate that impact in explicit budget allocations, leading to a concave pattern overall.

The study has limitations. While our moderate sample size is appropriate for identifying main fMRI effects, it is underpowered for testing individual differences, and the behavioural results should be treated with caution until replicated in larger samples. Furthermore, our sample was primarily left-leaning and non-isolationist, suggesting the observed home country bias may represent a conservative estimate. Different samples are needed to fully understand how these effects manifest in other populations. Real-world neural effects may also be larger or smaller than those observed here due to differences in context, information format, and the potential for demand characteristics in lab environments. For the willingness to pay task, the pattern of responses suggests the diminished marginal allocation reflects scope insensitivity rather than an effect of limited funds. Still, incentive-compatible replications with uncapped budgets would further bolster our understanding of these decisions.

These findings bridge neuroscience and global humanitarian challenges. The decline of neural responses with high numbers of victims and the diminished response to deaths abroad may be contributing to poor global outcomes in humanitarian disasters. Understanding these effects, alongside other biological, cognitive, and social constraints on our consideration of lives at risk, will be key to increasing engagement and saving lives.

## Supplementary Material

nsag049_Supplementary_Data

## Data Availability

All analysis scripts, statistical images and behavioural data used for this report are available at Figshare: https://doi.org/10.25377/sussex.30067162.
