# Supplementary material for: Diminished neural responses for greater numbers of lives at risk and lives lost in foreign countries
Source: Soc Cogn Affect Neurosci. 2026 Jul 8;21(1):nsag049. doi: 10.1093/scan/nsag049 (PMC13406401; doi:10.1093/scan/nsag049)
Supplement: nsag049_Supplementary_Data [file nsag049_supplementary_data.zip › Campbell-Meiklejohn_and_Cutler_Supplementary_Information.pdf]

Supplementary Information for:

Diminished Neural Responses for Greater Numbers of Lives at Risk and Lives Lost in Foreign Countries

This document includes:

Supporting text
Figures S1 to S3
Tables S1 to S17
Headlines Task Development, Validation, and Robustness

Daniel Campbell-Meiklejohn and Jo Cutler

Corresponding author: Dr. Daniel Campbell-Meiklejohn
Email: daniel.cm@sussex.ac.uk

Table of Contents

Instructions for the Willingness to Pay Task 3

Table S1. Countries of the Headlines Task 3

Table S2. The Headlines Task Conditions 4

Table S3. Post-scan Questions 4

Figure S1. Increase of Response with Number at Risk (all clusters). 5

Table S4. Activation Table. Increase of Response with Number at Risk. 6

Figure S2. Decrease of Response with Number of Home Country Lives at Risk (all clusters). 7

Table S5. Activation Table. Decrease of Response with Number of Home Country Lives at Risk. 7

Table S6. Activation Table. Differences in the Neural Response to the Number at Risk Between Home Country Lives and Financial Credits. 8

Table S7. Activation Table. Inverted-U Response to the Number of Lives at Risk Abroad. 9

Table S8. Direct Comparison of Response to Individual Life Headlines: Abroad < Home Country. 9

Figure S3. Lives Abroad at Risk > Home Country Lives at Risk. 10

Table S9. Activation Table. Lives Abroad at Risk > Home Country Lives at Risk. 10

Table S10. Region of Interest Analysis Multi-Level Linear Regression Table Predicting Dorsomedial Prefrontal Cortex Response from Location, Outcome, and Number of Lives 11

Table S11. Region of Interest Analysis Multi-Level Linear Regression Table Predicting Right Anterior Insula Cortex Response from Location, Outcome, and Number of Lives 11

Table S12. Region of Interest Analysis Multi-Level Linear Regression Table Predicting Left Anterior Insula Cortex Response from Location, Outcome, and Number of Lives 12

Table S13. Multi-level Linear Regression Tables Predicting ROI Responses from Location and Number of Lives, estimated separately for Dead and Alive outcomes. 13

Table S14. Activation Table. Home Country Lives, Dead > Alive. 14

Table S15. Activation Table. Location × Outcome Interaction. 14

Emotion and Surprise 14
Table S16. Multi-level Linear Regression Table for Emotion Rating 15
Table S17. Multi-level Linear Regression Table for Surprise Rating 16

Headlines Task Development, Validation, and Robustness 17
Pilot Ratings of Candidate Events 17
Cause Categorisation 18
Initial Analysis and Final Stimulus Set 18
Post Hoc Analysis and Checking Robustness at Outcome Stage 18

Instructions for the Willingness to Pay Task

"You must consider proposals and allocate funding. For each project, there is a maximum of £100,000 available which you should allocate if the project is very important. You must not spend unnecessary money because the money can also go to other worthy causes. If a project is not worthwhile, you can decide to give it £0. However, you cannot use the money you save on one project now for another one of the projects listed, imagine any money you save would be used on different future projects."

Table S1. Countries of the Headlines Task

Country	Number of Events	Country	Number of Events
The UK	100	France	1
America	7	Haiti	1
China	6	Iraq	1
India	6	Israel	1
Afghanistan	4	Italy	1
Nigeria	4	Jamaica	1
Syria	4	Jordan	1
Australia	3	Kenya	1
Chile	3	Kyrgyzstan	1
Japan	3	Laos	1
South Africa	3	Libya	1
Turkey	3	Mexico	1
Yemen	3	Mozambique	1
Bangladesh	2	Poland	1
Indonesia	2	Singapore	1
Nepal	2	Somalia	1
Pakistan	2	Spain	1
Romania	2	Sri Lanka	1
Sierra Leone	2	St Vincent	1
South Sudan	2	Sudan	1
Algeria	1	Sweden	1
Argentina	1	Taiwan	1
Botswana	1	Tanzania	1
Bulgaria	1	Thailand	1
Burma	1	The Philippines	1
Ecuador	1	The UAE	1
El Salvador	1	Uruguay	1
Eritrea	1	Venezuela	1
Estonia	1		

Table S2. The Headlines Task Conditions

Number bin	People				Credits	
	Home		Abroad			
	Alive (+)	Dead (-)	Alive (+)	Dead (-)	Keep (+)	Lose (-)
1	1 person		1 person		1 credit	
2	2 – 10 people		2 – 10 people		2 – 10 credits	
3	11 – 50 people		11 – 50 people		11 – 50 credits	
4	51 – 500 people		51 – 500 people		51 – 500 credits	
5	501 + people		501 + people		501 + credits	

Note. (+): positive outcome, (-): negative outcome.

Table S3. Post-scan Questions

Post-scan questions. Ratings on scale of does not describe me (1) to describes me very well (4)	*M*	*SD*
My UK national identity is important to me.	2.24	0.97
I identify strongly with the UK.	2.32	1.15
I really feel connected to the UK.	2.52	1.16
When bad things happen to other people in the UK it has an emotional impact on me.	3.04	1.14
When bad things happen to British people it has an emotional impact on me.	2.84	1.07
When bad things happen to other people in other countries it has an emotional impact on me.	3.20	0.91
When bad things happen to foreign people it has an emotional impact on me.	3.00	1.04
When good things happen to other people in the UK it has an emotional impact on me.	3.00	1.04
When good things happen to British people it has an emotional impact on me.	2.96	1.06
When good things happen to other people in other countries it has an emotional impact on me.	3.08	0.76
When good things happen to foreign people it has an emotional impact on me.	3.00	0.96
I worry more about bad things happening in the UK than abroad.	2.80	1.00
I worry more about bad things happening abroad than in the UK.	2.32	0.95
If I worried more about bad things happening in the UK than abroad, it would be because they are more likely to involve me.	3.20	1.16
If I worried more about bad things happening in the UK than abroad, it would be because they are more likely to involve people I know.	3.20	1.23
If I worried more about bad things happening in the UK than abroad, it would be because they are more likely to involve people I identify with.	2.60	1.23
I believe it's important to help people in the UK before helping people abroad.	2.12	1.36
I believe it's important to help people abroad before helping people in the UK.	2.00	0.91

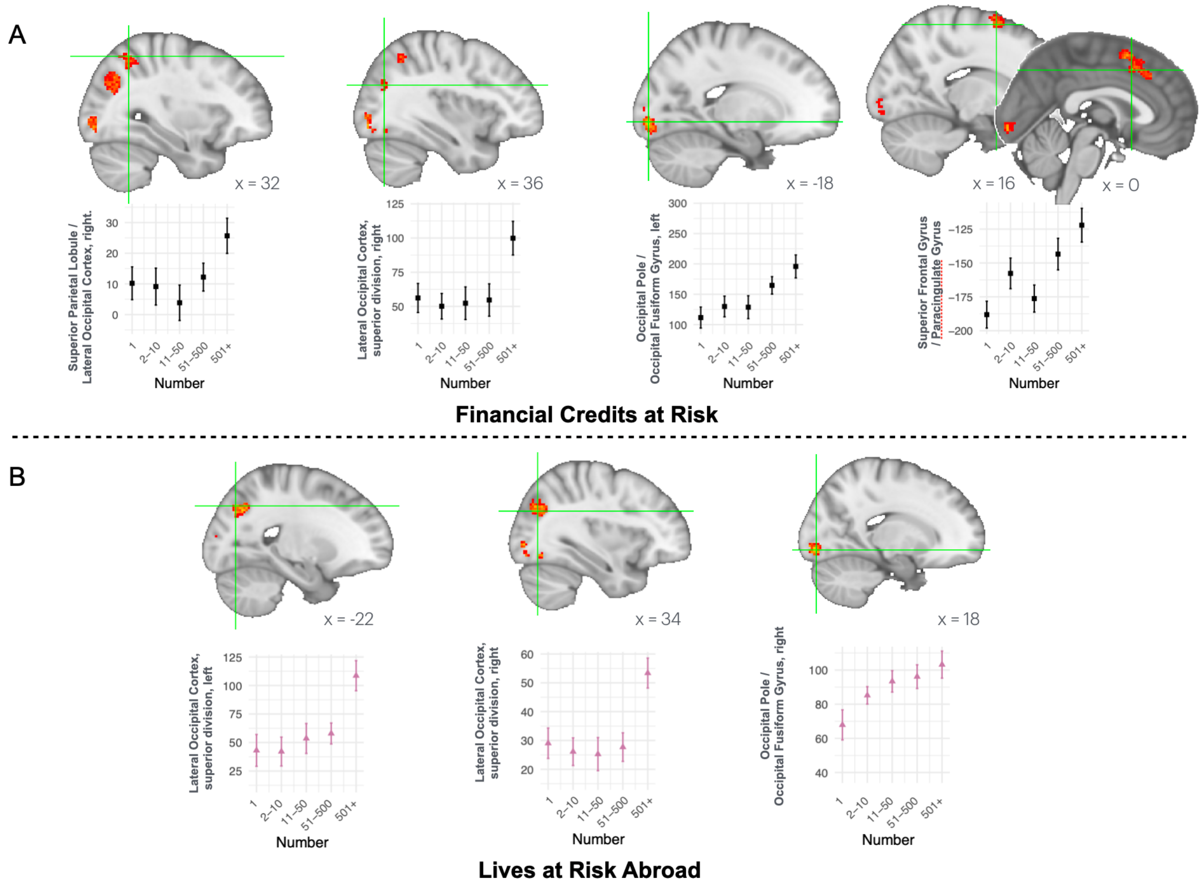

Figure S1. Increase of Response with Number at Risk (all clusters). Statistical maps show all significant clusters of increasing parametric response to the ($\log_{10}$) numbers of: A. financial credits at risk and B. lives abroad at risk ($Z > 3.1$, cluster $p < .05$). Plots below display average parameter estimates within each cluster, for each number bin. These are for visualisation purposes only. Error bars are within-subject standard error.

Table S4. Activation Table. Increase of Response with Number at Risk. Whole brain analysis results ($Z > 3.1$, cluster $p < .05$). Labels determined using the likelihood of voxel location in that region, using the Harvard-Oxford cortical and subcortical atlases. There was no increase for home country lives across numbers at risk. See Figure 3 and Figure S1.

Financial Credits at Risk

Cluster Index	Voxels	Z-MAX	X	Y	Z	Peak Location
4	783	4.8	-18	-92	-6	31% Occipital Pole,<br>9% Occipital Fusiform Gyrus
3	623	4.24	16	6	64	59% Superior Frontal Gyrus / dmPFC (subpeak)
2	175	4.26	36	-70	26	38% Lateral Occipital Cortex
1	118	4.16	32	-56	54	35% Superior Parietal Lobule,<br>21% Lateral Occipital Cortex

Lives at Risk Abroad

Cluster Index	Voxels	Z-MAX	X	Y	Z	Peak Location
3	259	4.02	-22	-74	40	54% Lateral Occipital Cortex,<br>2% Precuneous Cortex
2	255	4.19	18	-88	-8	33% Occipital Fusiform Gyrus,<br>19% Occipital Pole
1	242	4.03	34	-72	28	50% Lateral Occipital Cortex

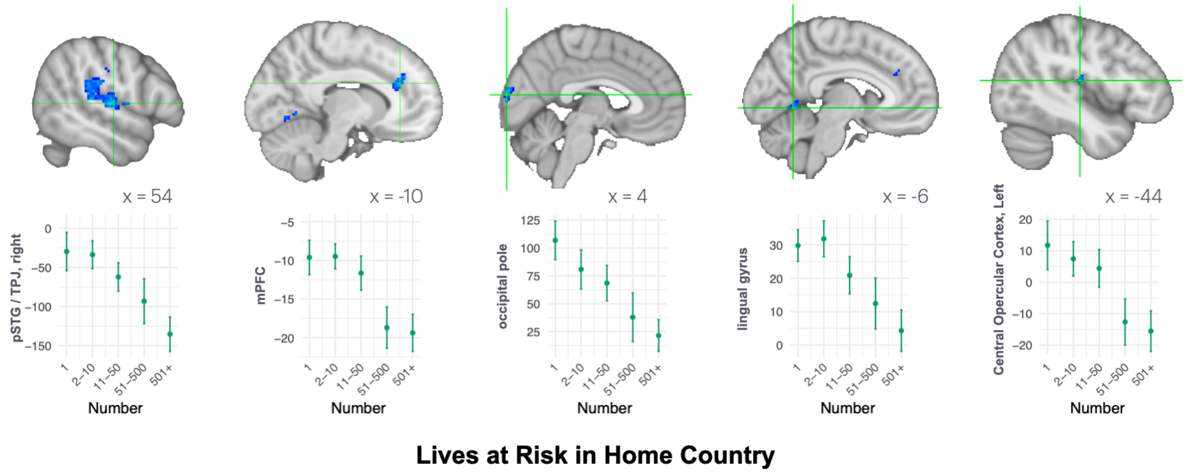

Figure S2. Decrease of Response with Number of Home Country Lives at Risk (all clusters). Statistical maps show all significant clusters of decreasing parametric response to the ($\log_{10}$) number of home country lives at risk ($Z > 3.1$, cluster $p < .05$). Plots below display average parameter estimates within each cluster, for each number bin. These are for visualisation purposes only. Error bars are within-subject standard error.

Table S5. Activation Table. Decrease of Response with Number of Home Country Lives at Risk. Whole brain analysis results (Z > 3.1, cluster p < .05). Labels determined using the likelihood of voxel location in that region, using the Harvard-Oxford cortical and subcortical atlases. See Figure S2.

Cluster Index	Voxels	Z-MAX	X	Y	Z	Peak Location
5	783	4.39	50	-28	12	52% Planum Temporale,<br>10% Parietal Operculum Cortex<br>(pSTG/TPJ)
4	351	4.22	4	-92	14	64% Occipital Pole,<br>9% Supracalcarine Cortex
3	124	3.94	-6	-60	-4	30%, Lingual Gyrus
2	90	3.94	-44	-18	14	65% Central Opercular Cortex,<br>12% Heschl's Gyrus
1	82	3.99	-10	34	24	39% Paracingulate Gyrus,<br>19% Cingulate Gyrus

Table S6. Activation Table. Differences in the Neural Response to the Number at Risk Between Home Country Lives and Financial Credits. Whole brain analysis results ($Z > 3.1$, cluster $p < .05$). Labels determined using the likelihood of voxel location in that region, using the Harvard-Oxford cortical and subcortical atlases. See Figure 4.

Cluster Index	Voxels	Z-MAX	X	Y	Z	Peak Location	Figure 4 Cluster Label
11	1337	4.49	-10	32	24	36% Paracingulate Gyrus, 23% Cingulate Gyrus	mPFC
10	253	4.5	0	-42	22	73% Cingulate Gyrus, 1% Precuneous Cortex	Posterior Cingulate Cortex
9	208	4.27	54	-32	22	41% Parietal Operculum Cortex, 24% Planum Temporale	pSTG/TPJ, right
8	164	4.31	-34	14	10	51% Frontal Operculum Cortex, 16% Insular Cortex	Anterior Insula, left
7	150	3.96	-4	-78	44	52% Precuneous Cortex, 12% Cuneal Cortex	Precuneus
6	145	4.06	18	10	66	60% Superior Frontal Gyrus	Superior Frontal Gyrus, right
5	111	4	34	20	-6	59% Insular Cortex, 11% Frontal Orbital Cortex	Anterior Insula, right
4	105	4	10	8	4	54% Right Caudate, 2% Right Lateral Ventricle	Ventral Caudate, right
3	99	3.99	-22	46	22	52% Frontal Pole	Frontal pole, left
2	90	4.02	-4	-94	8	63% Occipital Pole, 4% Cuneal Cortex	Occipital Pole
1	78	4.05	-18	-70	4	39% Intracalcarine Cortex, 9% Lingual Gyrus	Intracalcarine Cortex

Table S7. Activation Table. Inverted-U Response to the Number of Lives at Risk Abroad. Whole brain analysis results ($Z > 3.1$, cluster $p < .05$). Labels determined using the likelihood of voxel location in that region, using the Harvard-Oxford cortical and subcortical atlases. See Figure 5.

Cluster Index	Voxels	Z-MAX	X	Y	Z	Peak Location	Figure 5 Cluster Label
8	908	4.35	48	-32	18	38% Parietal Operculum Cortex, 31% Planum Temporale	pSTG/TPJ, right
7	401	4.31	40	6	4	36% Insular Cortex, 31% Central Opercular Cortex	Anterior insula, right
6	324	4.22	8	-24	42	66% Cingulate Gyrus, 7% Precentral Gyrus	Posterior Cingulate, right
5	307	4.3	-44	-22	64	51% Postcentral Gyrus, 12% Precentral Gyrus	Postcentral Gyrus
4	246	4.36	-14	-32	42	40% Precentral Gyrus, 19% Cingulate Gyrus	Posterior Cingulate, left
3	155	4.09	-38	-28	12	40% Heschl's Gyrus, 19% Planum Temporale	pSTG, left
2	141	3.98	-2	34	20	72% Cingulate Gyrus, 20% Paracingulate Gyrus	mPFC
1	139	3.99	44	-18	2	31% Heschl's Gyrus, 19% Planum Polare	STG, right

Table S8. Direct Comparison of Response to Individual Life Headlines: Abroad < Home Country. Whole brain analysis results ($Z > 3.1$, cluster $p < .05$). Labels determined using the likelihood of voxel location in that region, using the Harvard-Oxford cortical and subcortical atlases.

Cluster Index	Voxels	Z-MAX	X	Y	Z	Peak Location
7	207	4.01	12	-74	38	29% Precuneous Cortex, 25% Cuneal Cortex
6	179	3.96	6	-28	30	50% Cingulate Gyrus, 1% Cingulate Gyrus
5	173	3.96	54	12	10	52% Inferior Frontal Gyrus, 18% Precentral Gyrus
4	137	3.95	24	56	24	77% Frontal Pole
3	91	3.96	60	-48	26	59% Angular Gyrus, 17% Supramarginal Gyrus (right posterior TPJ)
2	89	3.92	6	36	32	66% Paracingulate Gyrus, 10% Superior Frontal Gyrus
1	87	3.96	0	14	36	67% Cingulate Gyrus, 9% Paracingulate Gyrus

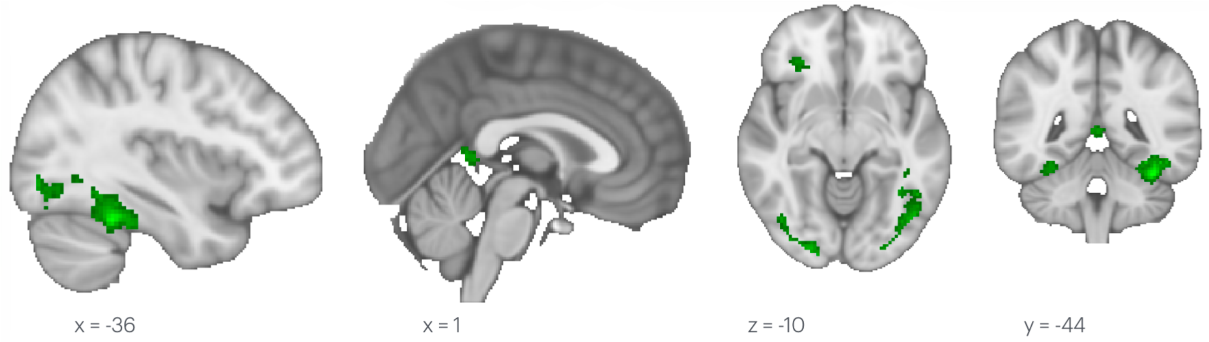

Figure S3. Lives Abroad at Risk > Home Country Lives at Risk. Statistical maps show clusters more active for lives at risk abroad than home country lives at risk, after controlling for the number at risk ($Z > 3.1$, cluster $p < .05$). Maps are overlaid on the standard MNI152 brain, with the coordinate of each slice shown in mm. Images are in radiological (R-L) orientation.

Table S9. Activation Table. Lives Abroad at Risk > Home Country Lives at Risk. Whole brain analysis results ($Z > 3.1$, cluster $p < .05$). Labels determined using the likelihood of voxel location in that region, using the Harvard-Oxford cortical and subcortical atlases. These are partial main effects of the model including $\log_{10}$(number of people affected).

Cluster Index	Voxels	Z-MAX	X	Y	Z	Location
5	1349	5.5	-36	-44	-24	43% Temporal Fusiform Cortex,<br>35% Temporal Occipital Fusiform Cortex
4	307	5.07	26	-84	-6	26% Occipital Fusiform Gyrus,<br>5% Occipital Pole
3	120	4.57	10	-54	12	53% Precuneous Cortex,<br>10% Intracalcarine Cortex
2	95	4.35	36	-36	-26	66% Temporal Fusiform Cortex,<br>17% Temporal Occipital Fusiform Cortex
1	90	4.25	28	32	-12	46% Frontal Orbital Cortex,<br>30% Frontal Pole

Table S10. Region of Interest Analysis Multi-Level Linear Regression Table Predicting Dorsomedial Prefrontal Cortex Response from Location, Outcome, and Number of Lives

Predictors	*B*	CI	*t*	*p*
Intercept	-53.17	-70.71 – -35.62	-5.95	**<.001**
Outcome [Dead > Alive]	7.83	0.98 – 14.68	2.25	**.025**
Log_{10}(Number of People)	0.36	-2.00 – 2.72	0.30	.765
Location [Abroad > Home Country]	0.73	-6.12 – 7.58	0.21	.834
Outcome × Log_{10}(Number of People)	6.16	1.44 – 10.88	2.56	**.011**
Outcome × Location	-15.73	-29.43 – -2.04	-2.26	**.024**
ICC	0.56			
$N_{SubjectID}$	25			
Observations	500			
Marginal R^2 / Conditional R^2	.015 / .565			

Best fitting model includes random intercept per participant.

Table S11. Region of Interest Analysis Multi-Level Linear Regression Table Predicting Right Anterior Insula Cortex Response from Location, Outcome, and Number of Lives

Predictors	*B*	CI	*t*	*p*
Intercept	-35.70	-44.78 – -26.63	-7.73	**<.001**
Outcome [Dead > Alive]	5.59	0.33 – 10.86	2.09	**.037**
Log_{10}(Number of People)	0.95	-0.87 – 2.77	1.03	.304
Location [Abroad > Home Country]	0.93	-4.34 – 6.19	0.35	.730
Outcome × Log_{10}(Number of People)	3.97	0.34 – 7.60	2.15	**.032**
Outcome × Location	-15.78	-26.31 – -5.25	-2.95	**.003**
ICC	0.35			
$N_{SubjectID}$	25			
Observations	500			
Marginal R^2 / Conditional R^2	.024 / .368			

Best fitting model includes random intercept per participant.

Table S12. Region of Interest Analysis Multi-Level Linear Regression Table Predicting Left Anterior Insula Cortex Response from Location, Outcome, and Number of Lives

Predictors	*B*	CI	*t*	*p*
Intercept	-15.24	-30.30 – -0.18	-1.99	**.047**
Outcome [Dead > Alive]	4.82	-0.75 – 10.39	1.70	.090
Log_{10}(Number of People)	0.72	-1.20 – 2.64	0.74	.460
Location [Abroad > Home Country]	-5.04	-10.61 – 0.53	-1.78	.076
Outcome × Log_{10}(Number of People)	3.91	0.07 – 7.75	2.00	**.046**
Outcome × Location	-16.57	-27.71 – -5.43	-2.92	**.004**
ICC	0.59			
$N_{SubjectID}$	25			
Observations	500			
Marginal R^2 / Conditional R^2	.016 / .592			

Best fitting model includes random intercept per participant.

Table S13. Multi-level Linear Regression Tables Predicting ROI Responses from Location and Number of Lives, estimated separately for Dead and Alive outcomes.

Dead Only

	**dmPFC**				**Right Anterior Insula**				**Left Anterior Insula**			
Predictors	*B*	*SE*	*t*	*p*	*B*	*SE*	*t*	*p*	*B*	*SE*	*t*	*p*
Intercept	-49.25	8.89	-5.54	**<.001**	-32.91	4.46	-7.38	**<.001**	-12.83	8.16	-1.57	.117
Location [Abroad > Home Country]	-7.13	5.06	-1.41	.160	-6.96	3.74	-1.86	.064	-13.32	4.09	-3.26	**.001**
Log_{10}(Number of People)	3.44	1.74	1.97	**.050**	2.93	1.55	1.90	.059	2.68	1.59	1.68	.093
ICC	0.53				0.34				0.60			
$N_{SubjectID}$	25				25				25			
Observations	250				250				250			
Marginal R^2 / Conditional R^2	.011 / .537				.022 / .353				.022 / .612			

Alive Only

	**dmPFC**				**Right Anterior Insula**				**Left Anterior Insula**			
Predictors	*B*	*SE*	*t*	*p*	*B*	*SE*	*t*	*p*	*B*	*SE*	*t*	*p*
Intercept	-57.08	9.18	-6.22	**<.001**	-38.50	5.14	-7.48	**<.001**	-17.65	7.35	-2.40	**.017**
Location [Abroad > Home Country]	8.60	4.80	1.79	.074	8.82	3.74	2.36	**.019**	3.24	3.91	0.83	.408
Log_{10}(Number of People)	-2.72	1.96	-1.39	.166	-1.03	1.29	-0.80	.424	-1.23	1.35	-0.91	.362
ICC	0.58				0.40				0.57			
$N_{SubjectID}$	25				25				25			
Observations	250				250				250			
Marginal R^2 / Conditional R^2	.010 / .589				.015 / .405				.003 / .569			

SE reported instead of *CI* due to space limitations.

Table S14. Activation Table. Home Country Lives, Dead > Alive. Whole brain analysis results ($Z > 3.1$, cluster $p < .05$). Labels determined using the likelihood of voxel location in that region, using the Harvard-Oxford cortical and subcortical atlases. See Figure 6C.

Cluster Index	Voxels	Z-MAX	X	Y	Z	Location
9	653	4.15	48	12	-8	38% Temporal Pole, 5% Insular Cortex
8	463	4.1	-44	18	-12	44% Frontal Orbital Cortex, 8% Temporal Pole
7	320	4.27	-8	28	38	39% Paracingulate Gyrus, 7% Superior Frontal Gyrus
6	287	4.09	4	12	62	61% Superior Frontal Gyrus,<br>10% Juxtapositional Lobule Cortex
5	272	4.31	-2	50	22	53% Paracingulate Gyrus, 15% Superior Frontal Gyrus
4	257	3.99	-42	-52	-22	55% Temporal Occipital Fusiform Cortex,<br>23% Inferior Temporal Gyrus
3	234	4.24	-8	2	12	53% Left Lateral Ventricle, 46% Left Caudate
2	194	3.96	-8	-76	-16	18% Lingual Gyrus, 9% Occipital Fusiform Gyrus
1	120	4.05	-24	-14	-24	86% Left Hippocampus, 1% Parahippocampal Gyrus

Table S15. Activation Table. Location × Outcome Interaction. Home Country Lives [Dead > Alive] > Lives Abroad [Dead > Alive]. Whole brain analysis results ($Z > 2.3$, cluster $p < .05$). Labels determined using the likelihood of voxel location in that region, using the Harvard-Oxford cortical and subcortical atlases. Subpeaks added for Cluster 6. See Figure 6D.

Cluster Index	Voxels	Z-MAX	X	Y	Z	Location
7	2234	3.71	26	-64	8	26% Intracalcarine Cortex, 9% Right Lateral Ventricle
6	706	3.8	-32	6	2	8% Left Putamen, 5% Insular Cortex
		3.42	-40	26	0	40% Frontal Operculum, 27% Frontal Orbital Cortex
		3.32	-34	24	4	40% Frontal Operculum, 32% Insular Cortex
5	640	3.95	-50	-34	-2	37% Middle Temporal Gyrus,<br>30% Superior Temporal Gyrus
4	551	3.5	8	-16	62	25% Precentral Gyrus,<br>12% Juxtapositional Lobule Cortex
3	439	3.37	32	26	-2	35% Insular Cortex,<br>27% Frontal Orbital Cortex
2	375	3.71	-42	-58	-12	35% Temporal Occipital Fusiform Cortex,<br>19% Inferior Temporal Gyrus
1	289	3	-48	30	14	48% Inferior Frontal Gyrus, 7% Middle Frontal Gyrus

Emotion and Surprise

We tested whether participants' self-reported ratings of emotion valence and surprise for each trial reflected the patterns of scope insensitivity and home country bias observed in the neural data. Ratings were analysed using linear mixed-effects models with Outcome, Location, and Log_{10}(Number of People) entered as mean-centred, effects-coded variables (±0.5).

For emotion valence, the model revealed expected main effects, including more negative ratings for death outcomes, events occurring abroad, and fewer victims (Table S16). The interaction of location and outcome only approached significance. The model also revealed a significant three-way interaction between Outcome, Log_{10}(Number of People), and Location. This indicates that the emotional divergence between survival and death scales across victim numbers depended on whether the event occurred in the home country or abroad.

Events were rated as more surprising when they involved larger numbers of people and when they occurred in the home country (Table S17). Significant two-way interactions indicated that the effect of victim number and the effect of death vs survival on surprise also depended on the event's location.

Table S16. Multi-level Linear Regression Table for Emotion Rating

	Emotion Rating			
**Predictors**	***B***	**CI**	***t***	***p***
Intercept	44.43	42.41 – 46.46	43.06	**<.001**
Outcome [Dead > Alive]	-34.54	-43.01 – -26.08	-8.00	**<.001**
Log_{10}(Number of People)	-1.11	-1.35 – -0.87	-9.04	**<.001**
Location [Abroad > Home]	0.87	0.20 – 1.54	2.53	**.011**
Outcome × Log_{10}(Number of People)	-3.93	-4.41 – -3.44	-15.97	**<.001**
Log_{10}(Number of People) × Location	-0.46	-0.94 – 0.02	-1.88	.060
Outcome × Location	1.14	-0.21 – 2.48	1.65	.098
Outcome × Log_{10}(Number of People) × Location	-2.55	-3.51 – -1.58	-5.18	**<.001**
ICC		0.09		
$N_{SubjectID}$		25		
Observations		5000		
Marginal R^2 / Conditional R^2		.521 / .563		

Best fitting model includes random intercept and random slope for outcome, grouped by participant.

Table S17. Multi-level Linear Regression Table for Surprise Rating

	Surprise Rating			
**Predictors**	***B***	**CI**	***t***	***p***
Intercept	41.93	37.28 – 46.58	17.67	**<.001**
Log_{10}(Number of People)	2.82	1.65 – 3.99	4.72	**<.001**
Location [Abroad > Home]	-4.59	-6.67 – -2.52	-4.34	**<.001**
Outcome [Dead > Alive]	-0.60	-3.08 – 1.89	-0.47	.638
Log_{10}(Number of People) × Location	-1.24	-2.05 – -0.43	-2.99	**.003**
Location × Outcome	-6.65	-8.92 – -4.38	-5.74	**<.001**
ICC		0.26		
$N_{SubjectID}$		25		
Observations		5000		
Marginal R^2 / Conditional R^2		.040 / .288		

Best fitting model includes random intercept and random slopes for outcome, location and log(number of people), grouped by participant.

Headlines Task Development, Validation, and Robustness

Pilot Ratings of Candidate Events

The Headlines Task (Figure 1) was originally designed for 120 trials, then later extended to 200. Ethics for this pilot was approved by the University of Sussex Sciences and Technology Cross schools research ethics committee: ER/JC620/7.

In Round 1, twenty-nine participants completed more than 90% of the items (27 completed 100% and 2 completed 92%) and were retained for analysis (14 female; age M = 28.86, SD = 12.74, n = 28 reporting age). We began with 156 headlines describing different potential causes of death assembled from real news stories and statistics published by reputable bodies. Each headline was formatted to isolate the cause of death (the number of people and country were stripped). The headline was then divided into two phases corresponding to the task events: a 'presentation' phase using the wording "X people were identified as at risk of death from [cause]", and an 'outcome' phase using the wordings "X people died from [cause]" and "X people survived [cause]" depending on what happened in the selected story. Duplicates were removed, leaving 89 unique causes of potential loss of life, and 110 outcome items (since 21 of the 89 causes led to both survival and death in different stories).

In Round 2, our aim was to increase the task to 200 headlines. 39 participants (33 female; age M = 19.1, SD = 0.69, range 18–20) all completed 100% of the survey. They rated the cause of death of a further 80 sourced stories, requiring 43 additional unique 'presentation phase' ratings and 44 additional death/survival outcomes.

Across both rating rounds, each statement was rated on three dimensions. Emotion was rated in response to "How emotional does this make you feel?" on a five-point intensity scale: Not at all emotional / Slightly emotional / Moderately emotional / Very emotional / Extremely emotional. Empathy was rated in response to "How much empathy do you feel for the people involved?" on the parallel five-point scale: Not at all empathic / Slightly empathic / Moderately empathic / Very empathic / Extremely empathic. Valence was rated in response to "How positive or negative is the emotion?" on a symmetric bipolar scale: Very negative / Slightly negative / Neither positive nor negative / Slightly positive / Very positive. For analysis, intensity-scale responses were coded 0–4 and valence responses −2 to +2; ratings were averaged across participants to produce a mean rating per cause on each dimension.

Combined ratings from both pilot rounds yielded full coverage of the 200 stimuli on the presentation phase and 199 of 200 on the outcome phase, with a single missing outcome rating ("a hurricane", ALIVE) imputed from the mean of the eight other natural-disaster ALIVE ratings.

Cause Categorisation

Each cause of death was assigned to one of five mutually exclusive categories that span the major sources of mortality represented in the corpus: Accidents (e.g., car crash, fire, plane crash); Diseases (e.g., cancer, HIV, Ebola); Human Violence (e.g., murder, terrorist attack, war); Natural disasters (e.g., earthquake, flooding, hurricane); and Long-term issues (e.g., poverty, hunger, suicide, malnutrition). These categories were used as a constraint during stimulus assembly to ensure that experimental conditions were matched on the broad type of cause depicted as well as on the rated affective dimensions.

Initial Analysis and Final Stimulus Set

The fMRI experiment used the final 200 news-headline stimuli arranged in 5 number bins (numbers of people: 1 / 2–10 / 11–50 / 51–500 / 501+) × 2 (location: home country / abroad) × 2 (outcome: alive / dead) factorial design, with 10 stimuli per cell across the 20 conditions. Stimuli were drawn from the rated set. The 200-stimulus set was balanced on broad cause category: each of the five categories contributed exactly 40 stimuli overall, exactly 8 stimuli to every number bin, and exactly 20 stimuli to each location condition (home and abroad). Each number bin × location cell also contained exactly 4 stimuli of every category, by design.

The task was designed on the basis that pilot presentation-phase affective responses to the cause of death ("X people were identified as at risk of death from [cause]") were balanced across number bins and location condition ("Location" variable in analysis). These ratings were tested using a categorical ANOVA model (number bin × Location) over the unique cause × number bin × Location combinations (n = 183; 17 cause × bin × Location combinations appear in both ALIVE and DEAD outcome versions, which share presentation-phase ratings). No effect approached significance on emotion, empathy, or valence (all $F \leq 1.99$, all $p \geq .16$, all $\omega^2 \leq .006$). The largest effect was a main effect of location on presentation valence ($F(1, 173) = 1.99$, $p = .160$, $\omega^2 = .006$), reflecting a small non-significant tendency toward more negative presentation valence for stories from the home country (M = −1.20) than stories from abroad (M = −1.15), but there were clearly no differences in cause ratings across number bins.

Overall, presentation-phase ratings of risk-to-life causes (e.g. "X people were identified as at risk of death from [cause]") showed no detectable linear or non-linear structure across the location or number conditions that could confound neural effects of these variables at the presentation stage of the task. The task was finalised on this basis.

Post Hoc Analysis and Checking Robustness at Outcome Stage

Since we were using real events, it was difficult to perfectly balance across location, number bin, and survival outcome simultaneously (20 conditions), even though the task was balanced across number bin and location at the presentation phase by design. Broad cause category counts ranged from 0 to 4 stimuli per cell (mean 2), reflecting a slight skew of some categories toward particular outcomes. Despite this finer-level variability, no condition cell departed strongly from the expected count of 2

(range 0–4); a chi-square test against uniform expectation was non-significant, χ^2 (76) = 50.00, p = .991, although with low expected counts, this test is conservative and is reported only as a coarse check. The question of whether an imbalance of cause categories across outcome types affects the rated affective properties of conditions is addressed by the analyses below.

Outcome-phase ratings from the pilot could not explain neural findings (i.e., the home-country bias to death vs survival, Figure 6, Tables S10-S12), but this conclusion required more consideration. Outcome-phase ratings were first analysed using the same regression model parameters as the primary region of interest analyses: Rating ~ Outcome + Log(Number of Lives) + Location + their two-way interactions, with Outcome and Location effect-coded (±0.5; Dead = +0.5; Abroad = +0.5) and Log(Number of Lives) mean-centred. As expected, the main effect of Outcome was large on all dimensions (all $p < .001$). On emotion and empathy intensity, no other effect was significant (all $p >$ .39). On the valence of experienced emotion, the only additional significant effect was Outcome × Location (B = -0.132, p = .015). This behavioural effect of the cause of death could not account for the home country bias in neural responses to 'dead' vs 'alive' (Figure 6, Tables S10-S12). The valence rating and neural interactions take very different shapes. The pilot-ratings of emotion valence for the outcome statements "X people died from [cause]" and "X people survived [cause]" differed markedly between survival and death outcomes regardless of whether they occurred at home or abroad, but outcomes abroad were slightly more polarised (alive vs dead valence differences of 2.20 (home) and 2.33 (abroad), on a 5-point scale). In contrast, the neural data showed a dead-vs-alive response difference for home-country lives that was absent for lives abroad. Valence differences from causes did not cause this neural result.

To confirm this statistically, we introduced emotion valence ratings as a covariate in our primary multi-level fMRI models of ROIs in Tables S10-S12. Emotion valence ratings were mean-centred within each Outcome condition, which allowed us to mathematically isolate the wider alive - dead valence gap for stories from foreign countries. Even when accounting for these subtle, stimulus-level fluctuations in subjective valence, the Location × Outcome interaction remained strongly significant across the dmPFC (p = .019), left anterior insula (p = .035), and right anterior insula (p = .004). Furthermore, the valence covariate itself was not a significant predictor of parameter estimates in any of the three ROIs. Similarly, entering pilot valence ratings of stories as covariates into the whole brain analysis of home-country bias (Figure 6C and 6D) did not change these results. Together, these findings robustly indicate that the differential neural processing of life-and-death outcomes between home countries and foreign countries operates independently of measured differences of affective valence from the *causes* of risk to life across the task.

Finally, consistent with our task design and presentation-phase validation, we searched for non-linear patterns of outcome-relevant ratings using ANOVAs with Number Bin coded as a 5-level categorical factor. These models detected trend-level Number Bin × Outcome interactions on outcome-

related emotion ($F(4, 180) = 2.32$, $p = .059$) and empathy ($F(4, 180) = 2.26$, $p = .064$), as well as a significant Number Bin × Location × Outcome interaction on outcome valence ($F(4, 180) = 4.02$, $p = .004$). These interactions indicate potential non-linear differences between conditions where the affective gap between death and survival potentially fluctuates slightly across group sizes. The wider alive-dead valence gap noted above for foreign country stories was not entirely uniform across bins, peaking when the headlines involved larger groups of 51–500 people. While these non-linear bin dynamics may be relevant for the future use and iterations of this task, our multi-level covariate models confirm that these stimulus-level valence fluctuations do not account for the Location × Outcome neural effects at the outcome stage of the task.

For further questions about this task, please contact Dr Dan Campbell-Meiklejohn, University of Sussex (daniel.cm@sussex.ac.uk)
